# Patient and healthcare professional engagement and time use within a randomised controlled trial: investigating intervention costs associated with remote person-centred care in Sweden

**DOI:** 10.1136/bmjopen-2025-099034

**Published:** 2025-10-09

**Authors:** Emmelie Barenfeld, Inger Ekman, Matilda Cederberg, Andreas Fors, Lilas Ali, Hanna Gyllensten

**Affiliations:** 1 Department of Health and Rehabilitation, University of Gothenburg Institute of Neuroscience and Physiology, Box 455, 405 30 Gothenburg, Sweden; 2 Institute of Health and Care Sciences, University of Gothenburg Sahlgrenska Academy, Gothenburg, Sweden; 3 University of Gothenburg Centre for Person-Centred Care (GPCC), Gothenburg, Sweden; 4 Department of Medicine, Geriatrics and Emergency Medicine, Sahlgrenska University Hospital Ostra Hospital, Gothenburg, Sweden; 5 Department of Psychotic Disorders, Sahlgrenska University Hospital, Gothenburg, Region Västra Götaland, Sweden; 6 Region Västra Götaland, Research, Education, Development and Innovation, Primary Health Care, Gothenburg, Sweden; 7 Department of Psychiatry, Sahlgrenska University Hospital, Gothenburg, Sweden

**Keywords:** Health economics, Health policy, Health Services for the Aged, Pulmonary Disease, Chronic Obstructive, Preventive Health Services, Person-Centered Care

## Abstract

**Objectives:**

To describe the usage patterns of patients and healthcare professionals (HCPs) using a person-centred telehealth and e-health intervention.

**Design:**

An exploratory, descriptive, observational study embedded in the “Person-centred care at a distance (PROTECT)” randomised controlled trial (ClinicalTrials.gov: NCT03183817) as part of a process evaluation. Data on intervention use and time spent on the intervention were collected. Descriptive statistics were calculated.

**Setting:**

Participants were recruited from nine public primary healthcare facilities located in various areas of Gothenburg, Sweden.

**Participants:**

110 patients participating in the intervention group in the PROTECT trial were included. Participants were diagnosed with chronic heart failure (CHF, n=42), chronic obstructive pulmonary disease (COPD, n=56) or both (n=12). They were 33–93 years old (mean 71 years).

**Primary and secondary outcome measures:**

A secondary outcome report on resource use.

**Intervention:**

The 6-month-long intervention was performed as an add-on to standard care and comprised person-centred telephone support and access to a digital platform. Per-protocol use included co-creation of a health plan via the telephone and use of the digital platform at least once. Forms of use were tailored to the preferences and needs of the patients.

**Results:**

Most intervention activities took place in the first 3 months of the intervention. Most patients used a combination of phone and digital support, spending most of their time using the digital platform. Overall, patients and HCPs spent 6 and 2.5 hours/patient using the intervention, respectively. Of this time, 1.5 hours involved synchronous communication through phone calls, with health-plan calls averaging 77 min.

**Conclusions:**

The intervention usage patterns of patients and HCPs differed. Despite HCPs being accessible when required, patients dedicated most of their time to self-care practices. Based on time distribution data, 15 full-time HCPs could potentially co-create, document and follow-up on health plans for 10 000 patients under study conditions.

**Trial registration number:**

ClinicalTrials.gov: NCT03183817.

**STRENGTHS AND LIMITATIONS OF THIS STUDY**

- Collected intervention use data from both patients and healthcare professionals.
- Detailed communication lists were regularly checked for accuracy.
- Imputations were used in order not to underestimate time use.
- Self-reported time on digital platform activities due to limited automatic registration.
- No assessment of impact on time use for self-management except on the digital platform.

## INTRODUCTION

 Effective use of healthcare resources is a top priority for decision-makers worldwide.^1^,^3^ Person-centred care is highlighted as a driver in reducing the cost of healthcare while maintaining or improving the quality of care.^4^ Effectively engaging in these practices requires healthcare professionals (HCPs) and patients to work in partnership and jointly plan care,^5^,^7^ which has been shown to promote patient engagement and self-management.^8^ This person-centred approach is also feasible in remote care,^9 10^ which is at present emphasised in international and Swedish policy documents.^2 3^ Remote interventions in this field (ie, e-health and telecare) to prevent health decline or promote health are thus a relatively new research area that merits further exploration from a resource use perspective, including the mapping of time use in interventions.^11^,^13^

Time use is a component of resource use that encompasses the cost of health services (eg, local expenses and staff salaries for working hours), costs associated with travel and education, and the time patients spend pursuing an intervention.^14^ Reports on the time use of patients and HCPs are requested in overall process evaluations of interventions^15^ and are essential to support the implementation of person-centred care.^13^ However, time spent on specific intervention activities is seldom detailed in economic evaluations.^15^ Studies have reported time use in person-centred care interventions,^1213 16^,^18^ but none of them evaluated remote interventions, which is a critical factor for controlling healthcare costs.^4^ In e-health, communication frequency and channels,^19^ as well as time spent by patients on healthcare activities after implementation,^20^ have been studied. Currently, there appears to be no research examining the time allocated to particular intervention activities within remote person-centred care.

Information on intervention use patterns is necessary to enhance decision-making, both in clinical practice and when considering changes to practice.^15 21 22^ Despite policy decisions to implement person-centred care across countries,^2 3^ there remains a lack of studies exploring what person-centred care entails in everyday clinical settings. Understanding how work in partnership between patients and HCPs may change workflows is crucial to support implementation, not least because it can be challenging, with concerns about resource demands and integration into existing care structures.^7 23^

This study is based on the randomised controlled trial (RCT) “Person-centred care at a distance (PROTECT)”,^24^ which evaluates an intervention meeting the European standard for person-centred care.^25^ The PROTECT intervention comprised a combination of telephone and digital support that focused on people diagnosed with chronic heart failure (CHF) and/or chronic obstructive pulmonary disease (COPD).^24^ While there was no effect on the primary outcome (a composite score of change in general self-efficacy and rehospitalisation or death 6 months after randomisation) in the PROTECT trial,^26^ patients perceived the intervention as a meaningful, accessible and appreciated support for initiating or maintaining self-management activities.^9 27^ The intervention was tailored to each individual’s needs and preferences, resulting in varying levels of use. Such tailoring, which is central to person-centred care,^5^,^7^ adds complexity to estimating resource use. Evaluating complex interventions benefits from integrating a health economic perspective in process evaluations, to address scalability, policy relevance and financial implications for uptake (eg, staffing requirements or costs incurred by patients in terms of dedicated time).^21 22^ Thus, this study has been undertaken to complement the health economic evaluation following the RCT PROTECT.^24^ The aim was to describe the usage patterns of patients and HCPs using a person-centred telehealth and e-health intervention.

Research questions:

How do patients and HCPs engage with the intervention?

At what time points and for how long time do patients and HCPs engage with the intervention?

## METHODS

### Design

This is an exploratory, descriptive, observational study nested in an RCT. The study complements the health economic evaluation with process outcomes by detailing intervention use along the RCT PROTECT^24^ (ClinicalTrials.gov: NCT03183817). Consolidated Health Economic Evaluation Reporting Standards 2022 (CHEERS 2022) Explanation and Elaboration was used.^28^

### Setting

The study included patients recruited from nine geographically distributed public primary healthcare centres in Gothenburg, Sweden, providing the opportunity to include patients with diverse sociodemographic characteristics. In the Swedish healthcare system, primary healthcare centres are expected to provide preventive services; however, such services are often downgraded in favour of treating health conditions of a more acute nature.^2^ Remote services and facilitation of self-care activities are promoted in national policy documents to encourage preventive actions.^2 29^

### Participants

The PROTECT study included 222 patients diagnosed with CHF and/or COPD (112 in the control group and 110 in the intervention group). The present study comprises all patients (n=110) allocated to the intervention group. Additional inclusion criteria in the main trial were: being listed at one of the participating primary care centres, understanding written and spoken Swedish, and having access to a device with an internet connection. Applied exclusion criteria were: severe impairment (eg, cognitive) preventing the use of the e-health support, lack of a registered address, expected survival of less than 12 months, an ongoing documented diagnosis of alcohol or drug abuse, other conditions that could interfere with follow-up, and participation in another conflicting study.^24^

### The PROTECT intervention

The PROTECT intervention^24^ was developed to support people diagnosed with CHF or COPD to work remotely with HCPs in the mutual planning of their care and treatment. The 6-month intervention comprised person-centred telephone support and access to a digital platform, in addition to usual care.^24^ This included various types of phone calls and digital platform activities, as outlined in table 1. Patients and HCPs co-created a health plan during their initial calls, and patients were recommended to engage in at least one scheduled follow-up call. The digital platform included functions such as messaging with HCPs, self-rating and symptom monitoring, reading or writing a personal health plan, and accessing relevant links. Patients could log in to the platform on their smartphones, tablets or computers. The intervention use was tailored to each participant’s needs and preferences, with the form of use mutually agreed on.^24^ Patients could contact HCPs during office hours over the phone or through the digital platform. Thus, per-protocol use included a minimum expected interaction, involving the co-creation of a health plan and the use of the digital platform.^24 26^

**Table 1:** Content of the phone calls and digital platform activities in the intervention

Phone calls	Description
Initial or follow-up health plan calls	Co-creation of a health plan (initial call) and follow-up of health plans.
Introduction to the digital platform	Guide to using telephone and digital support.
Technical support for the digital platform	Additional guidance on how to log in or use the platform or answer questions on the digital platform.
Healthcare support (additional to intervention)	Advice on health issues or medical questions.
**Digital platform activities**	
Messaging	Ability to contact HCPs around the clock (however, the chat was staffed only during office hours).
Shared health planning	The mutually agreed health plan was available to all involved partners: patients, their next of kin (if invited by patients) and HCPs.
Self-rating and monitoring symptoms and well-being	Three symptom ratings (breathing, sleep, tiredness) and two questions on well-being were included. The ratings were illustrated on a graph over time, visible to patients and HCPs. Patients could also write private notes.
Accessing information links	Links were included to validated information about CHF and COPD provided by patient organisations and the Swedish national health guide (1177.se), as well as to a peer-to-peer support group.

CHF, chronic heart failure; COPD, chronic obstructive pulmonary disease; HCPs, healthcare professionals.

HCPs conducting the intervention were located at a research unit and performed the intervention remotely. During the study period (2017–2021), the intervention team comprised five registered HCPs: three registered nurses, an occupational therapist and a physiotherapist. The number of project staff involved in patient-related work varied over time, ranging from 0.5 to 1 full-time equivalent, depending on the number of patients in the intervention. Any of HCPs involved could facilitate communication via phone calls and digital platforms. The intervention was designed to support partnerships by facilitating patients’ active engagement in their care and ensuring transparent documentation of all agreements, thereby enabling collaboration between different professionals. The training of HCPs performing the intervention is described elsewhere.^24^

### Routines for remotely implementing person-centred care

The present study incorporated three integrated routines to implement person-centred care: initiating, developing and safeguarding the partnership.^5 30^ The following section presents a brief description of how these routines were translated into practical actions during the PROTECT intervention.

#### Initiating the partnership

The key activities of HCPs to initiate the partnership involved treating the patient as a person with autonomy and resources (ie, being self-aware and able to take as much responsibility for the planning of care and treatment as desired)*.* The primary approach of the HCPs was to attentively listen to the patients’ experiences of their condition and how it affected their life*.*^5 30^ Dialogues between HCPs and patients can be either verbal or written.

#### Developing the partnership

A personal health plan was co-created based on patients’ narratives, the HCPs’ professional assessment and medical data. This plan was agreed on during the initial calls and served as a starting point for subsequent communication. In addition to discussing and agreeing on a health plan, communication included messaging on the digital platform, patients writing their health plan, and other telephone conversations between patients and HCPs (eg, technical support and healthcare support). Patients were also invited to rate their symptoms and health on the digital platform to monitor changes. The ratings could also be discussed during follow-up calls.

#### Safeguarding the partnership

Safeguarding the partnership encompassed documenting a written version of the health plan and uploading it to the digital platform, either by the patient or the HCP. The documentation contained a summary of the patient’s current situation, health goals, their internal and external resources for maintaining or improving health, their need for support to reach their goals, and the planned actions of the involved partners. The plan was regularly revised as needed.

### Data collection

Patient characteristics and demographics were collected from medical records and a baseline questionnaire. Intervention use and time spent on the intervention were obtained from the digital platform, communication lists developed by the project staff and a process-evaluation questionnaire sent by post after the 6-month intervention period. Data on the use of the digital platform were collected over a 24-month period.

The dates, number of messages, self-ratings and documented health plans were collected manually through the activities of each participant on the platform. Communication lists for each patient included date and type of contact (phone call, text message, e-mail), purpose of contact (introduction, technical support, healthcare support or scheduled health plan call), time spent (length of phone call), who initiated the contact, and whether it was planned or spontaneous. The length of each phone call was reported in minutes as registered on the telephone. Communication lists were updated after each call and quality-checked weekly by the HCPs’ team leader. HCPs reported the time spent on documenting health plans. The time that the patients spent on the digital platform (self-ratings, writing health plans and messaging) was collected through estimates in the process-evaluation questionnaire. Estimates of the time HCPs spent on checking the digital platform (eg, logging in to the system three times daily and checking for messages) and writing messages during the study were based on interviews with the HCPs’ team leader, who personally conducted most of these communications.

### Data management

Imputations were used in order not to underestimate time use. The reasons for the missing data guided the imputation choices, which were agreed on following discussions within the research team. The main reason for the missing data on the telephone support was a delay in implementing a more advanced data collection sheet. This sheet was designed to specify time use for various types of phone calls beyond health plan calls (eg, introduction, technical support and healthcare support calls). While the dates of these calls were documented from the outset of the study, their categorisation was conducted retrospectively for participants for whom the data sheet had not yet been developed, and time-use data had not been implemented. Details on the missing data, the applied data management and imputation method, and the motives for the choices are provided in online supplemental appendix 1.

Calculations of the time use for HCPs were converted to units of patients per full-time employee and full-time employee per 10 000 patients, based on the assumption that 1880 hours constitute one full-time year.^15^ We anticipated that approximately 10% of the work hours were spent on other tasks (eg, education, meetings and informal breaks). In calculations of the time use of HCPs, we assumed that the time spent checking the digital platform was equal regardless of the number of participants (ie, time used for taking action was measured separately).

### Data analysis

Descriptive analyses were calculated for patient characteristics and time spent on the digital platform (in minutes or hours). Continuous variables were summarised using mean, SD, median, minimum, maximum and IQR. Categorical variables were described by their counts and percentages. Analyses were conducted with IBM SPSS Statistics version 29 (IBM Corporation, Armonk, NY, USA).

### Patient and public involvement

Patient and public involvement partners were engaged in intervention development and the research process in the RCT PROTECT (eg, study design, interpretation of findings, and discussions on how to communicate and disseminate findings). Details on the co-creation of the intervention are provided elsewhere.^24^ In this study, a patient representative participated in discussions concerning the study design to ensure that the research questions reflected priorities from the patients’ perspective.

### Research ethics and participant consent

Ethical approval (refs: 063-17 and T063-18) was obtained from the Regional Ethics Review Board in Gothenburg, Sweden. Participants provided written informed consent.

## RESULTS

Demographic characteristics were comparable between the subgroup of participants who adhered to the intervention per protocol (n=76) and all individuals assigned to the intervention group according to the intention-to-treat principle (n=110), as detailed in table 2. Refer to online supplemental appendix 2 for the flowchart.

**Table 2:** Patient demographics and characteristics

	Intervention group	
	**Intention-to-treat** **n=110**	**Per-protocol group** **n=76**
Age		
Years, mean (SD)	71 (10)	70 (9)
Years, median (min, max)	72 (33–93)	71 (33–86)
Sex		
Women, n (%)	51 (46%)	31 (41%)
Civil status		
Living alone, n (%)	42 (38%)	27 (36%)
Diagnosis		
CHF, n (%)	42 (38%)	27 (36%)
COPD, n (%)	56 (51%)	38 (50%)
CHF and COPD, n (%)	12 (11%)	11 (14%)
Education level		
Compulsory, n (%)	38 (34%)	26 (34%)
Secondary school, n (%)	25 (23%)	18 (24%)
Vocational college, n (%)	25 (23%)	17 (22%)
University, n (%)	22 (20%)	15 (20%)
Self-rated technical competence		
Good or better, n (%)	57 (57%)*	46 (68%)†
Self-rated meaningful intervention use		
Yes, n (%)	53 (64%)‡	40 (68%)§
No, n (%)	12 (14%)‡	9 (15%)§
Don’t know, n (%)	18 (22%)‡	10 (17%)§
Intervention use		
Co-created a health plan, n (%)	108 (98%)	76 (100%)
Followed up on the health plan at least once, n (%)	106 (96%)	75 (99%)
Followed up the health plan on two or more occasions, n (%)	86 (78%)	61 (80%)
Per-protocol use¶, n (%)	76 (69%)	76 (100%)

* Missing n=15.

† Missing n=7.

‡ Missing n=27.

§ Missing n=17.

¶ Per-protocol use included at least one health plan call and use of the digital platform.

CHF, chronic heart failure; COPD, chronic obstructive pulmonary disease.

### Patterns of intervention engagement

Communication with HCPs by telephone was used by 109 patients (99%) at least once, compared with 76 patients (69%) who used the digital platform. Among those who used the digital platform, 47% (n=36) sent messages in the chat and 89% (n=68) used the self-rating tool (table 3). Of the patients using telephone support, 108 co-created a health plan with HCPs. All health plans were uploaded to the platform and the majority were evaluated in a follow-up call.

**Table 3:** Number of contacts per communication method for the intention-to-treat group (n=110) and for the per-protocol group (n=76)

	Intention-to-treat group n=110		Per-protocol group n=76	
	Number of contacts	Contacts per patient	Number of contacts	Contacts per patient
	Total contacts, (min-max)	Mean (SD); Median, Q1–Q3	Total contacts, (min-max)	Mean (SD); Median, Q1–Q3
Telephone support				
Health plan calls (co-creation and follow-up of health plan)	363, (0–6)	3.3 (1.0); 3, 3–4	261, (1–6)	3.4 (1.0); 3, 3–4
Introduction to the digital platform	109, (0–1)	1 (0.1); 1, 1–1	76, (0–1)	1 (0.0); 1, 1–1
Technical support of the digital platform	33, (0–3)	0.3 (0.7); 0, 0–0	25, (0–3)	0.5 (0.8); 0, 0–1
Healthcare support in addition to intervention	21, (0–5)	0.2 (0.6); 0, 0–0	15, (0–5)	0.2 (0.7); 0, 0–0
Digital platform use				
Messages, total	342, (0–17)	3.1 (3.8); 1, 1–4	313, (0–17)	4.1 (4.1); 3, 1–7
Messages written by patients	96, (0–8)	0.9 (1.7); 0, 0–1	96, (0–8)	1.3 (1.9); 0, 0–2
Messages written by HCPs	246, (0–9)	2.2 (2.3); 1, 1–3	217, (0–9)	2.9 (2.5); 2, 1–4
Self-ratings, total, months 1–24	2246, (0–526)	20.4 (63.0); 2, 0–7	2246, (0–526)	29.6 (74.0); 4, 1–17
Self-ratings, total, months 1–6	1394, (0–145)	12.7 (29.0); 1.5, 0–6	1394, (0–145)	18.3 (33.0); 3, 1–13
Shared documentation				
Health plan written by patients*	47, (0–4)	0.4 (0.9); 0, 0–0.3	46, (0–4)	0.6 (1.0); 0, 0–1
Health plan written by HCPs*	317, (0–6)	2.9 (1.3); 3, 2–4	216, (0–6)	2.8 (1.4); 3, 2–4
Other				
Email	44, (0–7)	0.4 (1.0); 0, 0–0	N/A	N/A
Booking and rebooking appointments	95†, (0–5)	0.9 (1.1)†; 1, 0–1	53‡, (0–5)	0.8 (1.1)‡; (1, 0–1)
SMS§	–	–	–	–

* One health plan was documented by both HCPs and the patient.

† Missing=12.

‡ Missing=6.

§ SMS was used, but all data are missing.

HCPs, healthcare professionals; N/A, Not applicable; SMS, Short Message Service.

In the digital platform user group, 30% of the messages (96/313) were written by patients. A little over one fourth (27%) of patients wrote or updated their health plan during the intervention. Overall, 13% of all health plans (47/364) were written by patients (table 3).

### Patterns of time use

**Figure 1:**
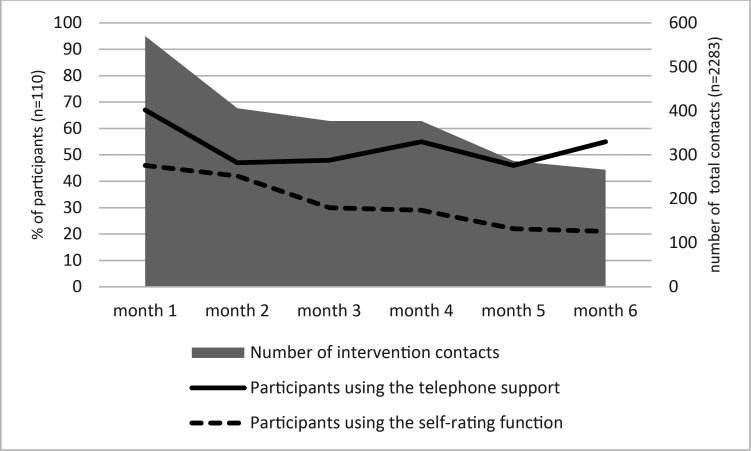
Proportion of participants using the telephone support and the self-rating function per intervention month, compared with the total number of intervention contacts per month.

Most of the contacts between patients and HCPs took place during the first 3 months of the intervention (figure 1). Most initial health plan calls occurred within the first month; whereas, follow-up call volume peaked during months 4 and 6. Usage patterns for many phone calls and activities on the digital support are detailed in online supplemental appendix 3. The use of the self-rating tool steadily declined during the intervention. Some digital platform users (n=13; 17%) continued to self-rate their health and symptoms after the intervention period of 6 months. Of the total number of self-ratings, 38% (852/2246) were performed during months 7–24.

The process of co-creating health plans involved an initial telephone call averaging 31 min (SD±10 min), followed by 2–3 additional brief calls per patient, with quartiles for total calls ranging from 3 to 4 per patient (see table 3). Additionally, patients received, on average, 15 min of telephone calls for introduction and technical support or healthcare support (table 4). The actual time each patient spent engaging in the overall telephone support varied between 10 and 309 min (mean: 92, median: 81) over the 6 months. However, most of the patients’ time was spent using the digital platform (on average 4.5 hours during the intervention, range: 0–60 minutes/week), resulting in a total time engaged with the intervention of 6 hours per patient (online supplemental appendix 4).

**Table 4:** Registered time for different conversations in the telephone support and healthcare professionals’ reported time of using the digital platform

	Actual time (minutes) per patient	Actual time (minutes) per occasion	Imputed time* (minutes) used for the study population n=110	Imputed time* (hours) per 10 000 patients
	Total; mean (SD), median (quartiles), range	Sum	Sum	Sum
Telephone support				
Health-plan calls (initial & follow-up) (363 calls for 108 people)	8321/108; 77 (41), 70 (50–98), 13–296	23	8475	12 841
Initial call (108 calls for 108 people)	*3300/108; 31 (10*), *28 (23–38*), *12–64*	*31*	*3361*	*5093*
Follow-ups of the health plan (255 calls for 106 people)	*5021/108; 46 (34*), *39 (23–60*), *0–232*	*20*	*5113*	*7748*
Introduction support of digital platform (109 calls for 109 people)	1132/109; 10 (4), 10 (10–10), 0–50	10	1142	1731
Technical support of digital platform (33 calls for 22 people)	348/108; 3 (10), 0 (0–4), 0–65	10	354	537
Healthcare support (21 calls for 14 people)	180/108†; 3 (11), 0 (0–0), 0–82	9	183	278
Overall telephone support	–	–	10 154‡	15 387
Digital platform activities				
Formulating and documenting health plans (317 plans for 108 people)	5405/108; 50 (29), 45 (34–65), 0–142	17	5505	8341
Sending messages and emails (386 messages for 110 people)	–	3	1158	1755

We have applied italics as Initial call and Follow-ups of the health plan are the two subgroups contributing to the total amount of Health-plan calls.

* In the scale up to 110 and 10 000 people, those never participating in the intervention were assumed to have a time use equal of the mean value for actual time per patient. This was assumed to not underestimate time use.

† Missing=1, see online supplemental appendix 1 for further information.

‡ Equals 1.5 hours/participant in the intention-to-treat group.

HCPs spent most of their time on health plan calls with patients and documenting health plans, averaging 2.5 hours per patient, with the following breakdown: health plan calls (77 min), other phone calls (15 min) and documentation (50 min) (table 4). The HCPs also spent approximately 78 hours per year checking the platform to assess whether further actions were needed (1.5 hours/week for all patients). Population size did not affect the time required to check the platform. Responding to messages from patients (n=110; 3 min/message) required an additional 19 hours. Translated into clinical practice, each HCP could provide the combined telephone and digital platform support to approximately 660 patients per year, equivalent to 15 full-time HCPs per 10 000 patients.

## DISCUSSION

Patients spent 6 hours and HCPs 2.5 hours per patient on the intervention, including 1.5 hours of joint phone calls. The findings show that a larger proportion of patients used telephone support than the digital platform (99% vs 69%), but the average time spent was higher for those using the digital platform. HCPs spent most of their time in the intervention interacting with patients through telephone support, followed by time spent documenting health plans. Typically, the phone calls related to health plans included a 30-min initial call conducted during the first month of the intervention, and 2–3 shorter follow-up calls distributed over the remaining 5 months, averaging 77 min per patient. Both HCPs and patients contributed to the health plan documentation and participated in platform messaging.

While HCPs spent most of their time in direct contact with patients via phone calls, patients spent most of their time using the digital platform as a self-management tool during the intervention. The report on time use for performing health plan calls aligns with secondary findings reported in an RCT evaluating telehealth for people diagnosed with CHF or COPD after hospital discharge.^31^ In contrast, less time use was reported compared with a similar, equally long intervention targeting people on sick leave due to common mental disorders.^32^ In our study, it was possible to further distinguish the time distribution between types of phone calls and intervention activities on the digital platform and time spent on documentation. Additionally, time use from a patient perspective contributed to the existing knowledge base. A chronic diagnosis often changes daily routines, requiring more time for self-care and healthcare interactions.^33^ While these activities are important to prevent health decline, they may also increase patients’ burden^34 35^ and should be considered in evaluations.^36^ Self-care is part of person-centred care and should be supported by work in partnership with HCPs.^25^ As stated by Jaarsma *et al*,^37^ two of the crucial underlying processes related to self-care are decision-making and reflection. Building on previous evaluations of the PROTECT intervention,^9 27^ our findings suggest that patients engaged in these processes both collaboratively with HCPs and independently. Considering the intervention activities, the time spent using the digital platform may likely overlap with activities that patients would undertake regardless of the intervention. At the same time, phone calls with HCPs are an addition usually not available in this form for the patient group. However, the lack of corresponding data in the RCT control group makes it impossible to assess how much of this is added time or whether it results in time savings elsewhere in self-care.

Our findings show that most patients (69%) employed a combination of telephone and digital support and remained engaged in the intervention for a large portion of the 6-month follow-up period. Identifying how people with chronic conditions want and can be involved in self-care activities to prevent further health decline is needed to achieve a sustainable healthcare system.^2 3^ Collaboration and understanding between patients and healthcare professionals—regarding self-care expectations, patient abilities and the need for support—are crucial for the effective management of CHF and COPD.^35 38^ An unexpected finding was that 27% of patients selected to document or revise their own health plan at least once, in consultation with HCPs. Our results suggest that some patients are willing to engage in formulating or updating their health plan, which merits further exploration into how this engagement can be supported. Sharing the agreed-upon health plan through documentation is a core activity in person-centred care.^5 7^ According to HCP reports, documenting and uploading the plan through a digital platform in the PROTECT intervention^24^ accounted for 50 min/patient during the intervention period. Although patients’ time was not detailed, it included multiple digital platform activities (Appendix 4). Having access to a health plan that supports self-care activities is a key quality marker in current health system redesigns.^2 3^ Our study could ascertain an approximate timeframe for the co-creation, documentation and subsequent follow-up of such plans.

The ability to access health-supporting innovations when needed is essential in enabling preventative efforts. Both remote services and person-centred care are important support structures in working towards this vision.^2 3^ In this study, the PROTECT intervention^24^ served as a case for exploring the intervention costs of remote person-centred care among individuals diagnosed with CHF or COPD. According to our findings, it appears that, based on the time distribution reported, it would be possible for 15 full-time HCPs to administer the intervention remotely to as many as 10 000 patients. The unique perspective gained from mapping time use and intervention engagement among both patients and HCPs can be used in strategic planning and decision-making processes, ultimately supporting the transition towards health-promoting and preventive services delivered through remote, person-centred care. When interpreting the results, it is essential to consider the characteristics of the study sample, which reflects the inclusion and exclusion criteria applied in the RCT. Moreover, the PROTECT intervention was offered as an add-on to usual care.^24^ Therefore, only usage costs related to the person-centred telehealth and e-health intervention are reported here. In contrast, usual care utilisation and costs will be presented in a currently ongoing health economic evaluation of PROTECT.^24^ Thus, our findings on time use and involvement in intervention activities, when combined with the health economic evaluation’s findings on healthcare resource use, can assist decision makers in determining the resources needed to provide person-centred care aimed at improving the health of people with chronic conditions, consistent with existing policy. In decision-making, these sources of evidence should be combined with evidence on the health outcomes of remote person-centred care interventions. This research area warrants further investigation into its efficacy.

### Strengths and limitations

A strength of this study was that data on time spent on telephone calls were recorded with precise information from the phone. Moreover, mapping different phone calls through detailed communication lists, which were regularly quality-checked by the team leader, could be considered a methodological strength. However, some methodological challenges were encountered in measuring time use. A major limitation was that the planned collection of login frequency and time required for activities from the digital platform could not be performed due to restrictions within the portal. Instead, digital platform activities were manually collected through the platform, and time spent on the platform was self-reported, which may have introduced bias (eg, memory or social desirability bias). Additionally, there were missing ratings of time spent on calls for technical and healthcare support for a small number of participants, primarily due to the initial communication lists not being fully developed at the trial’s outset. To handle missing data, either expert ratings from HCPs conducting the trial were used, or imputation based on time reported for other patients. Mean imputation can be used when data are missing completely at random,^39^ which was assumed here because the omissions were largely due to the delay in data collection at the start of the intervention. Despite the potential for bias resulting from this imputation method,^40^ it was necessary in this instance to prevent an underestimation of total time. To avoid the narrowing effect on confidence intervals caused by mean imputation, the intervals were reported based on pre-imputation data, while the imputed data were only added in the final estimate of total time.

Because the intervention was part of a research project, future studies are needed to explore time use while implementing remote person-centred care in clinical practice. For example, the time spent introducing new staff may lead to additional costs.^41^ We also did not evaluate how the intervention influenced the time patients spent on agreed-upon self-management activities, which is considered a methodological limitation, as most time spent in self-care occurs outside the healthcare system.^42^

## CONCLUSIONS

This study provides insights into how usage patterns of a remote person-centred intervention differed between patients and HCPs. Although HCPs were accessible as needed, patients predominantly devoted their time to self-care activities. Given the reported time distribution in the PROTECT trial, a team of 15 full-time HCPs could co-create, follow-up, and document health plans and be accessible for health support regarding these plans to 10 000 patients. This finding should be interpreted considering the characteristics of the study sample, which was determined by the inclusion and exclusion criteria applied in the RCT. While our observations indicate potential for scaling up remote person-centred care in preventive services, further investigation is needed to assess the effectiveness of these interventions in improving health outcomes. In addition, their costs should be evaluated within real-world settings and across more diverse patient populations.

## Supplementary material

10.1136/bmjopen-2025-099034 online supplemental appendix 1

## Data Availability

Data are available upon reasonable request.
