# Supplementary material for: Patient and healthcare professional engagement and time use within a randomised controlled trial: investigating intervention costs associated with remote person-centred care in Sweden
Source: BMJ Open. 2025 Oct 9;15(10):e099034. doi: 10.1136/bmjopen-2025-099034 (PMC12519657; doi:10.1136/bmjopen-2025-099034)
Supplement: online supplemental appendix 1 [file bmjopen-15-10-s001.docx]

Online-Only Supplements

**Authors**

Emmelie Barenfeld^1,2,3*^ Associate professor
Inger Ekman^1,3,4^ Senior Professor
Matilda Cederberg^1,3,5^ PhD
Andreas Fors^1,3,6^ Professor
Lilas Ali^1,3,7^ Associate professor
Hanna Gyllensten^1,3^ Associate professor

**Author affiliations**

^1^ University of Gothenburg, Sahlgrenska Academy, Institute of Neuroscience and Physiology, Department of Health and Rehabilitation, Gothenburg, Sweden

^2^ University of Gothenburg, Sahlgrenska Academy, Institute of Health and Care Sciences, Gothenburg, Sweden

^3^ University of Gothenburg Centre for Person-Centred Care (GPCC), Sahlgrenska Academy, University of Gothenburg, Sweden

^4^ Sahlgrenska University Hospital/Östra, Department of Medicine, Geriatrics and Emergency Medicine, Gothenburg, Sweden

^5^ Sahlgrenska University Hospital, Department of Psychotic Disorders, Gothenburg, Sweden.

^6^ Region Västra Götaland, Research, Education, Development and Innovation, Primary Health Care, Gothenburg, Sweden

^7^ Sahlgrenska University Hospital, Department of Psychiatry, Gothenburg, Sweden

| **Content** |  | **Page** |
| --- | --- | --- |
| Appendix 1:  Appendix 2: | Overview of imputation methods and choices.  Flowchart over participants included in the analysis. | 2  3 |
| Appendix 3:  Appendix 4: | Overview of total number of activities per intervention participant and the distribution of contacts per intervention month for different types of phone calls and ways to interact on the digital platform.  Self-reported time using the digital platform/week and imputed sum of minutes/week among patients (n=76) who logged in to the platform. | 4  5 |

**Appendix 1. Overview of missing data guiding data management and imputation choices.**

| **Type of resource use** | **Reported activities and time** | **Handling of missing data*** |
| --- | --- | --- |
| **Telephone support** | | |
| Health plan calls | Registered in logbook | No missing data.  Two participants in the intervention group had no health plan calls, thus zero time use. |
| Introduction to the digital platform and intervention | Activities registered in logbook  Time: A standard value of 10 minutes applied throughout the study; deviations from this value were recorded in logbook | One participant in the intervention group had no introduction call, thus zero time use. |
| Technical support  (optional type of telephone call) | Registered in logbook | No missing data. |
| Health care support (non-interventional)  (optional type of telephone call) | Registered in logbook | One participant in the intervention group had a registered health care support call without an indicated time use; for this person, the mean time per such call (9 minutes) were imputed. |
| **Digital platform use and emails**  (optional activities) | | |
| Digital platform use HCPs | Template time (1.5 hours per week for HCPs) for keeping an eye on the digital platform, based on assessment by HCP’s team leader  (Additional time use as listed for messages and emails) |  |
| Digital platform use patients | Self-reported activities and time from questionnaire at 6 months and 12 months, includes time for self-ratings, messages, accessing the health plan, reading information etc. | If not reporting 6-month data, 12-month data were used.  Mean time (15 min) imputed for 14 patients where activities were reported but no associated time.  Eleven participants had not used the platform, thus zero time use. |
| *Messages and emails* | Activities manually collected from digital platform and the projects email  Time (3 minutes per activity) based on assessment by research nurse |  |
| *Self-ratings* | Activities manually collected from digital platform  (No separate time listed) | **NA** |
| Health plans written by patients | Registered in logbook |  |
| Health plans written by HCPs | Registered in logbook |  |

NA = Not applicable
*In the scale up to 110 and 10 000 people those never participating in the intervention were assumed to have a time use equal of the mean value for each time use. This was assumed to not underestimate time use.

**Appendix 2. Flowchart over participants included in the analysis**

|  | Consented to participation in the PROTECT trial  n=222 |  |  |  |
| --- | --- | --- | --- | --- |
|  |  |  | Allocated to control group  n=112 |  |
|  | Allocated to intervention group n=110 |  |  | **Intention to treat analysis; n=110** |
|  |  |  | No contact n=1 |  |
|  | Received introduction to digital platform  n=109 |  |  |  |
|  |  |  | Declined intervention  n=1 |  |
|  | Co-created at least one health plan n=108 |  |  |  |
|  |  |  | No digital platform use n=32 |  |
|  | Used the digital platform n=76 |  |  | **Per-protocol analysis; n=76** |

**Appendix 3. Overview of total number of activities per intervention participant and the distribution of contacts per intervention month for different types of phone calls and ways to interact on the digital platform.**

|  | **Month 1** | **Month 2** | **Month 3** | **Month 4** | **Month 5** | **Month 6** | **Month 7+** |
| --- | --- | --- | --- | --- | --- | --- | --- |
| **Telephone support** | | | | | | | |
| Health plan calls n=363 | 73 (20%) | 55 (15%) | 52 (14%) | 58 (16%) | 53 (14.6%) | 62 (17%) | 10 (2.8%) |
| Introduction to the digital platform and intervention n=109 | 109 (99%) | 0 (0%) | 0 (0%) | 0 (0%) | 0 (0%) | 0 (0%) | 0 (0%) |
| Technical support n=33 | 11 (33%) | 9 (27%) | 6 (18%) | 4 (12%) | 2 (6%) | 0 (0%) | 1 (1%) |
| Health care support (non-interventional) n=21 | 3 (14%) | 5 (24%) | 2 (10%) | 3 (14%) | 4 (19%) | 3 (14%) | 1 (5%) |
| Total contacts telephone support/intervention month n=525 | 196 (37%) | 69 (13%) | 59 (11%) | 65 (12%) | 59 (11%) | 65 (12%) | 12 (2%) |
| **Digital platform use & emails** | | | | | | | |
| Messages and emails n=363^a^ | 162 (43%) | 79 (24%) | 49 (13%) | 34 (9%) | 21 (6%) | 18 (5%) | - ^a^ |
| Self-ratings n=1394^b^ | 212 (15%) | 245 (18%) | 270 (19%) | 279 (20%) | 206 (14%) | 182 (13%) | - ^b^ |
| Total contacts digital support/intervention month  n=1757^a b^ | 374 (21%) | 324 (18%) | 319 (18%) | 313 (18%) | 227 (13%) | 200 (11%) | - ^a, b^ |
| **Overall contacts with intervention** | | | | | | | |
| Number of contacts/intervention month n=2283 | 570 (25%) | 406 (18%) | 378 (16%) | 377 (16%) | 286 (12%) | 266 (12%) | 12 (1%) |
| Corresponds to number of contacts/participant in the intention to treat group | 5.2 | 3.7 | 3.4 | 3.4 | 2.6 | 2.4 | 0.1 |

^a^ missing n=13, post-interventional messages (n=10) not included, ^b^ post-interventional self-ratings (n=852) not included.

**Appendix 4. Self-reported time using the digital platform/week and imputed sum of minutes/week among patients (n=76) who logged in to the platform.**

| Total time in minutes spent per week ^a^ | 933 |
| --- | --- |
| Mean (SD) ^a^ | 15 (15) |
| Median (Q1-Q3) ^a^ | 10 (5–21) |
| Range ^a^ | 0–60 |
| Imputed sum total minutes per week ^b^ | 1143 |

^a^ missing n=14 (i.e. statistics are for n=62 patients), ^b^ imputed to cover all 76 patients.
