# Supplementary material for: Patient and healthcare professional engagement and time use within a randomised controlled trial: investigating intervention costs associated with remote person-centred care in Sweden
Source: BMJ Open. 2025 Oct 9;15(10):e099034. doi: 10.1136/bmjopen-2025-099034 (PMC12519657; doi:10.1136/bmjopen-2025-099034)
Supplement: Supplementary file 2 [file bmjopen-2025-099034.reviewer_comments.pdf]

PEER REVIEW HISTORY

BMJ Open publishes all reviews undertaken for accepted manuscripts. Reviewers are asked to complete a checklist review form and are provided with free text boxes to elaborate on their assessment. These free text comments are reproduced below.

ARTICLE DETAILS

Title (Provisional)

Patient and healthcare professional engagement and time use within a randomised controlled trial: Investigating intervention costs associated with remote person-centred care in Sweden

Authors

Barenfeld, Emmelie; ekman, inger; Cederberg, Matilda; Fors, Andreas; Ali, Lilas; Gyllensten, Hanna

---

VERSION 1 - REVIEW

**Reviewer**	**1**
**Name**	**Flink, Maria**
**Affiliation**	**Karolinska Institutet, Neurobiology, Care Sciences and Society**
**Date**	**12-Jun-2025**
**COI**	**None**

Thank you for the opportunity to review this well-written and interesting study reporting on the process evaluation of the PROTECT trial.

Methods:

Regarding the intervention, please clarify if the patients' contact with HCP was with a specific person or anyone among the professionals, and if the HCPs were part of the patient's regular professionals or a new contact. If anyone among the professionals and new contact, how did this correspond to safeguarding the partnership?

Data analysis. Minor detail: please explain the abbreviations ITT and PP.

Results:

Table 3. I would suggest removing the "Telephone support n=525" and "Digital platform use n=2588" etc. It is a bit confusing since these numbers refer to the first column with the 110 participants.

“Most of the communications were initiated by the HCPs.” It is unclear to me why this information is important. Was this communication pattern not according to the intervention as you write “Forms for communication were mutually agreed upon”?

Discussion and conclusion

I overall lack a discussion on how this process evaluation can support the interpretation of the effect study’s results. As the RCT did not have any significant effect, the main focus for me of a process evaluation would be to understand why the intervention did not produce any effect. I interpret your low patient and HCP engagement (6 resp 2.5 hours over 6 months) as a clear signal that the patients did not actually receive the intervention. However, your aim does not indicate that this is a process evaluation, rather that you use data from the RCT to report on time used in digital interventions. If so, you should consider removing “process evaluation” from your title.

I think that the statement “Our findings show that with the reported time distribution, 14 full-time HCPs could provide the PROTECT remote intervention to 10 000 patients. This points towards the potential for the intervention to be expanded to larger groups of people diagnosed with CHF and COPD.” may be misleading. As I understand reference 22, your RCT did not show significant differences between groups at your endpoint, which I interpreted as potentially resulting from the in this study identified low patient and HCP engagement. I would suggest rephrasing the above statement and the conclusion so that it is not misinterpreted as a signal that PROTECT had an effect and should be expanded to larger groups.

Checklist:

How come you choose CHEERS as your reporting guideline checklist? This study is not a health economic evaluation.

**Reviewer**	**2**
**Name**	**Hewison, Jenny**
**Affiliation**	**University of Leeds, Leeds Institute of Health Sciences**
**Date**	**21-Jul-2025**
**COI**	**None**

The goal of making care more person-centred (PCC) is an admirable one, and at the level of principle, requires no further justification. If however there is a need to justify the effort spent in promoting it in terms of clinical or psychological benefit to patients, or increased efficiency of the healthcare system, then caution is required. This paper describes supplementary analyses to a previously reported effectiveness RCT in which

the intervention was the introduction of a number of changes to care, aimed at making it more person centred, and the primary outcome was ‘a composite score of change in general self-efficacy and hospitalization or death 6 months after randomization’. In this RCT, no difference on the primary outcome measure was found between intervention and control arms. There is an understandable desire to defend the

principle of PCC in the light of results such as these. RCTs are not infallible: they can only recruit people content with the care in either arm, interventions and measures of benefit have to be operationalised in particular ways, and the participants following a protocol will differ from those who do not. A descriptive process analysis can contribute to the evidence base around what PCC might mean, how it might be promoted, and the effects on patients that might – and might not - result. Unfortunately, the authors of this

paper have instead tried to use their process analysis to find some positive messages about the benefits of their version of PCC, concluding that, ‘the intervention has the potential to increase support for self management in people diagnosed with COPD or CHF.’ A lot of effort has been expended to find this message, and the conclusion is unconvincing as a result. There are also more specific problems with the paper, of

which the most serious is the comparison of the per-protocol group with the intervention group as a whole, rather than with non-compliers. Pearson’s chi-square is a test for independence, and by definition, these two groups are clearly not independent. Using a p-value ≤ 0.05 multiple times to indicate a statistically significant

result is also not good practice, and a number of the imputation decisions do not withstand careful scrutiny. A final piece of advice to the authors is more clearly to distinguish their PROTECT trial from the very famous ProtecT three-arm RCT in prostate cancer.

VERSION 1 - AUTHOR RESPONSE

Comments from reviewers	Response	Reference to revisions
**Reviewer 1**		
Thank you for the opportunity to review this well-written and interesting study reporting on the process evaluation of the PROTECT trial.	Thank you for taking the time to review the manuscript and for your encouraging feedback. We appreciate your thoughtful comments and are pleased to hear that you found the study well-written and interesting.	
Methods:<br>Regarding the intervention, please clarify if the patients’	We have clarified that the follow-up calls and digital platform contacts could involve any of the	**Methods**<br>The PROTECT intervention<br>2nd<br>paragraph

contact with HCP was with a specific person or anyone among the professionals, and if the HCPs were part of the patient's regular professionals or a new contact.	professionals in the intervention team.<br><br>The HCPs were not part of the patients regular professional contacts. We have clarified this by rephrasing a paragraph in the methods section.	HCPs conducting the intervention were located at a research unit and performed the intervention remotely. During the study period (2017–2021), the intervention team comprised five registered HCPs: three registered nurses, an occupational therapist, and a physiotherapist. The number of project staff involved in patient-related work varied over time, ranging from 0.5 to 1 full-time equivalent, depending on the number of patients in the intervention. Any of HCPs involved could facilitate communication via phone calls and digital platforms. The intervention was designed to support partnerships by facilitating patients' active engagement in their care and ensuring transparent documentation of all agreements, thereby enabling collaboration between different professionals. The training of HCPs performing the intervention is described elsewhere.[24]
If anyone among the professionals and new contact, how did this correspond to safeguarding the partnership?	Thank you for this important observation. The concern regarding continuity and the potential impact of new professional contacts on work in partnership is well taken. In line with the foundations of PCC the partnership is not solely dependent on individual continuity, but rather on the consistent application of person-centred care across all encounters. This includes initiating the partnership by active listening to patients´narratives, working the partnership by mutual agreement on activities and goals, and securing the partnership by documenting this. Thus, while the introduction of new professionals may pose a challenge to relational continuity, the intervention aimed to safeguard the partnership by embedding person-centred principles into the	**Methods**<br>The PROTECT intervention<br>2nd paragraph<br>See text above please.

	care process itself. We have clarified this aspect in the methods section.	
Data analysis. Minor detail: please explain the abbreviations ITT and PP.	We have deleted this sentence from the methods section.	**Methods**<br>~~Participant characteristics and demographics were compared between the ITT and PP groups using Pearson's chi-square test for categorical variables, with a p-value ≤ 0.05 used to indicate a statistically significant result.~~
Results: Table 3. I would suggest removing the "Telephone support n=525" and "Digital platform use n=2588" etc. It is a bit confusing since these numbers refer to the first column with the 110 participants.	Thank you for your helpful suggestion. We have removed the numbers as recommended to improve clarity in Table 3. We have also made clarifications in Table 4 according this feedback.	See **Table 3** and Table 4
"Most of the communications were initiated by the HCPs." It is unclear to me why this information is important. Was this communication pattern not according to the intervention as you write "Forms for communication were mutually agreed upon"?	We have deleted the text as suggested.	**Results**<br>Patterns of intervention engagement<br>2nd paragraph<br><br>~~Most of the communications were initiated by the HCPs. Patients accounted for 5% (n=28) of all phone calls, mainly related to technical support and non-interventional healthcare support.~~
Discussion and conclusion:<br>I overall lack a discussion on how this process evaluation can support the interpretation of the effect study's results. As the RCT did not have any significant effect, the main focus for me of a process evaluation would be to	We understand and appreciate your point. That the intervention had no significant effects and was limited apart from the three-month follow-up in the per-protocol analysis has been stated in the introduction (4th paragraph) and is also briefly discussed in the manuscript to which we refer. It is indeed important to reflect on how process-evaluation	**Title**<br>Patient and healthcare professional engagement and time use within a randomised controlled trial: Investigating intervention costs associated with remote person-centred care<br><br>**Abstract**<br>Design: An exploratory, descriptive, observational study embedded in the "Person-centred care at a distance (PROTECT)" randomised controlled trial (ClinicalTrials.gov: NCT03183817) as part of a process evaluation.<br><br>**Methods**<br>Design

understand why the intervention did not produce any effect. I interpret your low patient and HCP engagement (6 resp 2.5 hours over 6 months) as a clear signal that the patients did not actually receive the intervention.	findings relate to the outcomes of the RCT. However, according to our research questions at the end of the introduction of this manuscript the primary aim of this part of the process evaluation was not to further explain the lack of significant effect of the main outcome, but rather to contextualize the health economic evaluation by exploring the practical implications of implementing a remote person-centred intervention (eg. describing the workflow), with a specific focus on resource use and associated costs. Our study aligns with the broader understanding of process evaluation as outlined in the updated MRC framework (Skivington et al., 2021), which includes examining resource use, economic implications, and how the intervention integrates into existing workflows. By focusing on these aspects, our aim was to generate insights that can inform if and how similar interventions could be practically implemented and scaled up in routine care. This has now been clarified throughout the manuscript by rephrasing, adding or removing text to better highlight the focus.<br><br>It is also worth noting that a separate, yet unpublished, health economic evaluation	This is an exploratory, descriptive, observational study nested in an RCT. The study complements the health economic evaluation with process outcomes by detailing intervention use along the RCT PROTECT [24](ClinicalTrials.gov: NCT03183817). Consolidated Health Economic Evaluation Reporting Standards 2022 (CHEERS 2022) Explanation and Elaboration was used.[29]<br><br>**Introduction**<br>3rd paragraph<br>4th paragraph<br><br>Information on intervention use patterns is necessary to enhance decision-making, both in clinical practice and when considering changes to practice.[15 21 22] Despite policy decisions to implement person-centred care across countries[2 3] there remains a lack of studies exploring what person-centred care entails in everyday clinical settings. Understanding how work in partnership between patients and HCPs may change workflows is crucial to support implementation, not least because it can be challenging, with concerns about resource demands and integration into existing care structures[7 23].<br><br>This study is based on the randomised controlled trial (RCT) "Person-centred care at a distance (PROTECT)", [24] which evaluates an intervention meeting the European standard for person-centred care[25]. The PROTECT intervention comprised a combination of telephone and digital support that focused on people diagnosed with chronic heart failure (CHF) and/or chronic obstructive pulmonary disease (COPD).[24] While the intervention had a limited effect on the primary outcome in the PROTECT-trial[26] patients perceived the intervention as a meaningful, accessible, and appreciated support for initiating or maintaining self-management activities.[9 27] The intervention was tailored to each individual's needs and preferences, resulting in varying levels of use. Such tailoring, which is central to person-centred care[5-7], adds complexity to estimating resource use. Evaluating complex interventions benefits from integrating a health economic perspective in process evaluations, to address scalability, policy relevance, and financial implications for uptake (e.g. staffing requirements or costs incurred by patients in terms of dedicated time)[21 22]. Thus, this study has been

	shows a beneficial impact of the intervention on societal costs and health-related quality of life, further supporting the relevance of understanding resource use. If the evaluation is accepted for publication, we will be able to include it as a reference.	undertaken to complement the health economic evaluation following the RCT PROTECT.[24] The aim was to describe the usage patterns of patients and HCPs using a person-centred telehealth and e-health intervention.<br><br>**Discussion**<br>4th paragraph<br><br>The ability to access health-supporting innovations when needed is essential in enabling preventative efforts. Both remote services and person-centred care are important support structures in working towards this vision.[2 3] In this study, the PROTECT intervention[24] served as a case for exploring the intervention costs of remote person-centred care to promote health and prevent health decline among individuals diagnosed with CHF or COPD. According to our findings, it appears that, based on the time distribution reported, it would be possible for 15 full-time HCPs to administer the intervention remotely to as many as 10 000 patients. The unique perspective gained from mapping time use and intervention engagement among both patients and HCPs can be used in strategic planning and decision-making processes, ultimately supporting the transition towards health-promoting and preventive services delivered through remote, person-centred care. When interpreting the results, it is essential to consider that the PROTECT intervention was offered as an add-on to usual care.[24] Therefore, only usage costs related to the person-centred telehealth and e-health intervention are reported here. In contrast, usual care utilisation and costs will be presented in a currently ongoing health economic evaluation of PROTECT.[24] Thus, our findings on time use and involvement in intervention activities, when combined with the health economic evaluation's, findings on healthcare resource use, can assist decision makers in determining the resources needed to provide person-centred care aimed at improving the health of people with chronic conditions, consistent with existing policy. In decision-making, these sources of evidence should be combined with evidence on the health outcomes of remote person-centred care interventions. This research area warrants further investigation into its efficacy.
However, your aim does not indicate that	We understand your concern and have revised	**Title**<br>Please see previous reply.

this is a process evaluation, rather that you use data from the RCT to report on time used in digital interventions. If so, you should consider removing "process evaluation" from your title.	the title accordingly to better reflect the focus of the study.	
I think that the statement "Our findings show that with the reported time distribution, 14 full-time HCPs could provide the PROTECT remote intervention to 10 000 patients. This points towards the potential for the intervention to be expanded to larger groups of people diagnosed with CHF and COPD." may be misleading.<br><br>As I understand reference 22, your RCT did not show significant differences between groups at your endpoint, which I interpreted as potentially resulting from the in this study identified low patient and HCP engagement. I would suggest rephrasing the above statement and the conclusion so that it is not misinterpreted as a signal that PROTECT had an	Thank you for your comment.<br>We have rephrased the statement and the conclusion, so that it is not misinterpreted as a signal that PROTECT impacted on the main outcome and should be expanded to larger groups. Moreover, we have been rewriting parts of the discussion to clarify how decision making in health care is influenced by several aspects, such as intervention evidence, policies and cost effectiveness. We also highlight how our findings can contribute to informed decision-making regarding person-centred approaches, in line with current policies on implementing person-centred care.<br><br>We understand your interpretation that the lack of significant effects of the main outcomes in the RCT and agree that the level of engagement is an important aspect to consider. Please see our previous reply on our	**Discussion**<br>The end of the 3rd paragraph<br>Our study could ascertain an approximate timeframe for the co-creation, documentation, and subsequent follow-up of such plans.<br><br>4th paragraph<br>The ability to access health-supporting innovations when needed is essential in enabling preventative efforts. Both remote services and person-centred care are important support structures in working towards this vision.[2 3] In this study, the PROTECT intervention[24] served as a case for exploring the intervention costs of remote person-centred care to promote health and prevent health decline among individuals diagnosed with CHF or COPD. According to our findings, it appears that, based on the time distribution reported, it would be possible for 15 full-time HCPs to administer the intervention remotely to as many as 10 000 patients. The unique perspective gained from mapping time use and intervention engagement among both patients and HCPs can be used in strategic planning and decision-making processes, ultimately supporting the transition towards health-promoting and preventive services delivered through remote, person-centred care. When interpreting the results, it is essential to consider that the PROTECT intervention was offered as an add-on to usual care.[24] Therefore, only usage costs related to the person-centred telehealth and e-health intervention are reported here. In contrast, usual care utilisation and costs will be presented in a currently ongoing health economic evaluation of PROTECT.[24] Thus, our findings on time use and involvement in intervention activities, when combined with the health economic evaluation's, findings on healthcare resource use, can assist decision makers in determining the resources needed to provide person-centred care aimed at improving the health of people with chronic

effect and should be expanded to larger groups.	chosen perspective in this study.	conditions, consistent with existing policy. In decision-making, these sources of evidence should be combined with evidence on the health outcomes of remote person-centred care interventions. This research area warrants further investigation into its efficacy.<br><br>**Conclusion**<br>~~The usage patterns of the intervention varied between the patients and the HCPs. Although the HCPs were accessible as required, the patients predominantly invested their time in self-management activities. This observation suggests that the intervention holds the promise of expanding support for self-management among persons diagnosed with CHF or COPD. In a practical context, a team of 14 full-time HCPs could effectively implement the intervention to 10 000 patients. Future research is warranted to determine the optimal timing for initiating the intervention.~~<br>This study provides insights into how usage patterns of a remote person-centred intervention differed between patients and HCPs. Although HCPs were accessible as needed, patients predominantly devoted their time to self-care activities. Given the reported time distribution in the PROTECT trial, a team of 15 full-time HCPs could co-create, follow up, and document health plans and be accessible for health support regarding these plans to 10 000 patients. This observation suggests that there may be room for scaling up remote person-centred care in preventive services. Nonetheless, further investigation is needed to assess the effectiveness of these interventions in improving health outcomes, as well as to determine their costs within the framework of real-world settings.
Checklist:<br>How come you choose CHEERS as your reporting guideline checklist? This study is not a health economic evaluation.	We agree that no single reporting guideline fully captures the nature of our study. While this study is not a full health economic evaluation, it serves as a complement to one. Given our focus on resource use and practical implementation aspects, we found the CHEERS checklist to be the most relevant framework	n.a

	available to guide transparent reporting.	
**Comments from reviewers**	**Response**	**Reference to revisions**
**Reviewer 2**		
	Thank you for your thoughtful and constructive review. Your comments have helped us reflect more critically on our interpretation of the findings. We also appreciate your methodological feedback. Below, we address each of your specific comments in detail.	
The goal of making care more person-centred (PCC) is an admirable one, and at the level of principle, requires no further justification. If however there is a need to justify the effort spent in promoting it in terms of clinical or psychological benefit to patients, or increased efficiency of the healthcare system, then caution is required.	We agree that person-centred care should already be an integral part of healthcare in countries where policies mandate its implementation. The manuscript has been revised accordingly by clarifying that we aim to address a knowledge gap by contributing data on what PCC entails in practice, specifically in terms of time use and intervention engagement. As the intervention applied general working principles to operationalize PCC based on the European standard entitled Patient involvement in healthcare-Minimum requirements for person-centred care (2020), these insights can support decision-making and inform the design of future interventions and studies. We acknowledge that our approach is normative, reflecting the policy context in several countries where working	**Introduction**<br>3rd and 4th paragraph<br><br>Information on intervention use patterns is necessary to enhance decision-making, both in clinical practice and when considering changes to practice.[15 21 22] Despite policy decisions to implement person-centred care across countries[2 3] there remains a lack of studies exploring what person-centred care entails in everyday clinical settings. Understanding how work in partnership between patients and HCPs may change workflows is crucial to support implementation, not least because it can be challenging, with concerns about resource demands and integration into existing care structures[7].<br><br>This study is based on the randomised controlled trial (RCT) "Person-centred care at a distance (PROTECT)", [24] which evaluates an intervention meeting the European standard for person-centred care[25]. The PROTECT intervention comprised a combination of telephone and digital support that focused on people diagnosed with chronic heart failure (CHF) and/or chronic obstructive pulmonary disease (COPD).[24] While the intervention had a limited effect on the primary outcome in the PROTECT-trial[26] patients perceived the intervention as a meaningful, accessible, and appreciated support for initiating or maintaining self-management activities.[9 27] The intervention was tailored to each individual's needs and preferences, resulting in varying levels of use. Such tailoring, which is central to person-

	in a person-centred way is expected, although implementation remains highly variable.<br><br>We have also been rewriting the discussion and conclusion and highlighted the need of future effectiveness studies of person-centred care interventions.	centred care[5-7], adds complexity to estimating resource use. Evaluating complex interventions benefits from integrating a health economic perspective in process evaluations, to address scalability, policy relevance, and financial implications for uptake (e.g. staffing requirements or costs incurred by patients in terms of dedicated time)[21 22]. Thus, this study has been undertaken to complement the health economic evaluation following the RCT PROTECT.[24] The aim was to describe the usage patterns of patients and HCPs using a person-centred telehealth and e-health intervention.<br><br>Below are changes made to the specific comment. For the whole paragraph and the conclusion please see previous reply to reviewer 1.<br><br>**Discussion**<br>4th paragraph<br>In this study, the PROTECT intervention[24] served as a case for exploring the intervention costs of remote person-centred care to promote health and prevent health decline among individuals diagnosed with CHF or COPD. According to our findings, it appears that, based on the time distribution reported, it would be possible for 15 full-time HCPs to administer the intervention remotely to as many as 10 000 patients.<br><br>When interpreting the results, it is essential to consider that the PROTECT intervention was offered as an add-on to usual care.[24] Therefore, only usage costs related to the person-centred telehealth and e-health intervention are reported here. In contrast, usual care utilisation and costs will be presented in a currently ongoing health economic evaluation of PROTECT.[24] Thus, our findings on time use and involvement in intervention activities, when combined with the health economic evaluation's, findings on healthcare resource use, can assist decision makers in determining the resources needed to provide person-centred care aimed at improving the health of people with chronic conditions, consistent with existing policy. In decision-making, these sources of evidence should be combined with evidence on the health outcomes of remote person-centred care interventions. This research area warrants further investigation into its efficacy.<br><br>**Conclusion**

		This study provides insights into how usage patterns of a remote person-centred intervention differed between patients and HCPs. Although HCPs were accessible as needed, patients predominantly devoted their time to self-care activities. Given the reported time distribution in the PROTECT trial, a team of 15 full-time HCPs could co-create, follow up, and document health plans and be accessible for health support regarding these plans to 10 000 patients. This observation suggests that there may be room for scaling up remote person-centred care in preventive services. Nonetheless, further investigation is needed to assess the effectiveness of these interventions in improving health outcomes, as well as to determine their costs within the framework of real-world settings.
This paper describes supplementary analyses to a previously reported effectiveness RCT in which<br>the intervention was the introduction of a number of changes to care, aimed at making it more person centred, and the primary outcome was 'a composite score of change in general self-efficacy and hospitalization or death 6 months after randomization'. In this RCT, no difference on the primary outcome measure was found between intervention and control arms. There is an understandable desire to defend the principle of PCC in the light of results such as these. RCTs are not infallible: they can only recruit people content with	Thank you for your thoughtful reflection on the challenges of interpreting null findings in complex intervention trials. We agree that RCTs, while valuable, have inherent limitations—particularly in how interventions and outcomes must be operationalized, and in the characteristics of participants who adhere to protocols. As you note, these factors can complicate interpretation, especially when results are inconclusive.<br>Our aim with this supplementary analysis is not to defend the principle of PCC, but rather to explore how the intervention was used in practice and what insights this may offer about implementation and engagement in person-centred care. We have clarified this purpose more explicitly in the introduction and removed one paragraph in the	**Introduction**<br>3rd paragraph<br>4th paragraph<br><br>Please see previous reply<br><br>**Discussion**<br>~~Our findings showed that most of the patients (69%) utilised a combination of telephone and digital support and were engaged in the intervention over a large portion of the 6-month follow-up period. Moreover, 17% of the digital platform users chose to continue with self-ratings after the intervention, indicating that there was a desire for continued use for some participants. An essential intervention mechanism reported in the qualitative evaluations of the PROTECT RCT[8 23] and in other studies evaluating tele- or e-health solutions[9 33 34] was the availability of trustworthy and competent HCPs through a direct dial number or digital support. One potential risk that was raised was that e-health interventions could create a feeling of dependence between patients and staff,[35] potentially resulting in unnecessary healthcare use. In contrast, the present study showed a trend of decreased intervention use over time, indicating that a few phone calls and access to the platform were sufficient to support patients with their main concerns. There was a low occurrence of spontaneous contacts from patients, with a negligible amount of additional time spent by HCPs for unplanned healthcare (i.e., providing health advice and guidance to health services). There often exists a general resistance from HCPs to give patients direct access, but expectations of unnecessary calls were contradicted by previous research. Instead, physicians who shared their private phone~~

the care in either arm, interventions and measures of benefit have to be operationalised in particular ways, and the participants following a protocol will differ from those who do not.	discussion of the revised manuscript to ensure that this is not interpreted as a defense.	~~number with patients were perceived to be more caring and received very few calls.[36 37]~~
A descriptive process analysis can contribute to the evidence base around what PCC might mean, how it might be promoted, and the effects on patients that might – and might not - result. Unfortunately, the authors of this paper have instead tried to use their process analysis to find some positive messages about the benefits of their version of PCC, concluding that, 'the intervention has the potential to increase support for self management in people diagnosed with COPD or CHF.' A lot of effort has been expended to find this message, and the conclusion is unconvincing as a result.	Thank you for highlighting the importance of maintaining a descriptive and exploratory focus in process evaluations. We agree that our original framing may have overemphasized positive interpretations with a focus on the PROTECT intervention. In response, we have revised the discussion and conclusion to better reflect the descriptive nature of the analysis and to present the findings more cautiously, acknowledging both the potential and the limitations of the intervention. We have also reframed the introduction to clarify expected contributions of the study in a broader context of relevance for decision makers. Moreover, we have rephrased the description of the design and the intervention to clarify our focus. We have also added references to other PCC frameworks when introducing PCC in the background and in the discussion.	**Introduction**<br>3rd paragraph<br>4th paragraph<br><br>Please see previous reply<br><br>**Methods**<br>Design<br>This is an exploratory, descriptive, observational study nested in an RCT. The study complements the health economic evaluation with process outcomes by detailing intervention use along the RCT PROTECT [24](ClinicalTrials.gov: NCT03183817). Consolidated Health Economic Evaluation Reporting Standards 2022 (CHEERS 2022) Explanation and Elaboration was used.[28]<br><br>The PROTECT intervention deleted text<br>~~The hypothesis was that working in partnership through a digital platform and telephone support would be a feasible and effective use of resources, leading to reduced hospital admissions and increased self-efficacy.~~<br><br>**Discussion**<br>1st, 2nd, 3rd and 4th paragraph<br><br>Patients spent 6 hours and HCPs 2.5 hours per patient on the intervention, including 1.5 hours of joint phone calls. The findings show that a larger proportion of patients used telephone support than the digital platform (99% vs. 69%), but the average time spent was higher for those using the digital platform. HCPs spent most of their time in the intervention interacting with patients through telephone support, followed by time spent documenting health plans. Typically, the phone calls related to health plans included a 30-minute initial call conducted during the first month of the intervention, and 2–3 shorter follow-up calls distributed over the remaining 5 months, averaging 77 minutes per patient. Both HCPs and patients

		contributed to the health plan documentation and participated in platform messaging.<br><br>While HCPs spent most of their time in direct contact with patients via phone calls, patients spent most of their time using the digital platform as a self-management tool during the intervention. The report on time use for performing health plan calls aligns with secondary findings reported in an RCT evaluating telehealth for people diagnosed with CHF or COPD after hospital discharge [31] In contrast, less time use was reported compared to a similar, equally long intervention targeting people on sick leave due to common mental disorders.[32] In our study it was possible to further distinguish the time distribution between types of phone calls and intervention activities on the digital platform and time spent on documentation. Additionally, time use from a patient perspective contributed to the existing knowledge base. A chronic diagnosis often changes daily routines, requiring more time for self-care and healthcare interactions. [33] While these activities are important to prevent health decline, they may also increase patients' burden [34 35] and should be considered in evaluations.[36] Self-care is part of person-centred care and should be supported by work in partnership with HCPs.[25] As stated by Jaarsma et al, [37] two of the crucial underlying processes related to self-care are decision-making and reflection. Building on previous evaluations of the PROTECT intervention,[9 27] our findings suggest that patients engaged in these processes both collaboratively with HCPs and independently. Considering the intervention activities, the time spent using the digital platform may likely overlap with activities that patients would undertake regardless of the intervention. At the same time, phone calls with HCPs are an addition usually not available in this form for the patient group. However, the lack of corresponding data in the RCT control group makes it impossible to assess how much of this is added time or whether it results in time savings elsewhere in self-care.<br><br>For paragraph 3 and 4 please see previous reply.<br><br>**Conclusion**<br>Please see previous reply<br><br>**References**<br>6. McCormack B, McCance TV. Development of a framework for

<table>
<tr>
<td></td>
<td></td>
<td>person-centred nursing. Journal of Advanced Nursing 2006;56(5):472-79. doi: 10.1111/j.1365-2648.2006.04042.x
7. Santana MJ, Manalili K, Jolley RJ, et al. How to practice person-centred care: A conceptual framework. Health expectations : an international journal of public participation in health care and health policy 2018;21(2):429-40. doi: 10.1111/hex.12640</td>
</tr>
<tr>
<td>There are also more specific problems with the paper, of which the most serious is the comparison of the per-protocol group with the intervention group as a whole, rather than with non-compliers.</td>
<td>Thank you for your comment.
We understand your concern regarding the comparison between the per-protocol group and the full intervention group. However, our intention with the analysis was not to explain differences in outcomes between those who complied with the protocol and those who did not to explain results. Instead, we selected these two groups to explore and illustrate variations in resource use and engagement with the intervention as a complement to the health economic evaluation. We have added a flowchart of participants included in the analysis.

Both the Intention to treat (ITT) and per-protocol (PP) analyses are included to offer complementary insights. The ITT analysis may underestimate the time required across a full population, particularly if standard care successfully ensures active participation from all individuals. Conversely, the PP analysis may overestimate time</td>
<td>Methods
Data analysis
~~Participant characteristics and demographics were compared between the ITT and PP groups using Pearson's chi-square test for categorical variables, with a p-value ≤ 0.05 used to indicate a statistically significant result.~~

Results
1st paragraph
~~No statistically significant differences in characteristics or demographics were found between the intervention group as a whole and those who followed the intervention protocol.~~

Table 2. Patient demographics and characteristics.
<table>
<tr><td></td><td colspan="2">Intervention group</td></tr>
<tr><td></td><td>~~Intervention group~~ Intention-to-treat group n=110</td><td>Per-protocol group n=76</td></tr>
</table>

Table 3. Number of contacts per communication method for the ~~intervention~~ intention-to-treat group (n=110) and for the per-protocol group (n=76).
<table>
<tr><td></td><td colspan="2">~~Intervention group~~ Intention-to-treat group N=110</td><td colspan="2">Per-protocol group N=76</td></tr>
<tr><td></td><td>number of contacts</td><td>contacts per patient</td><td>number of contacts</td><td>contacts per patient</td></tr>
<tr><td></td><td>total contacts, (min-max)</td><td>mean (SD) Median, Q1-Q3</td><td>total contacts, (min-max)</td><td>mean (SD) Median, Q1-Q3</td></tr>
</table>

Appendix 1; Flowchart</td>
</tr>
</table>

	requirements when scaling up, especially if the distribution of active participants remains similar or decreases at the population level.<br><br>Reviewer feedback has made us aware that this intention was not sufficiently clear in the original manuscript. Therefore, we have removed text from the methods section to clarify this as well as in the presentation of results. Moreover, we have revised the text throughout the manuscript to clarify the focus of the study.-Please also see our earlier response to Reviewer 1 regarding the scope of the process evaluation.	**Appendix 1. Flowchart over participants included in the analysis**<br>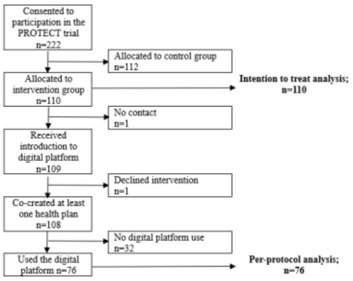
Pearson's chi-square is a test for independence, and by definition, these two groups are clearly not independent.<br>Using a p-value ≤ 0.05 multiple times to indicate a statistically significant result is also not good practice,	Thank you for your comment. We acknowledge the problems with using Pearson's chi-square test in this context. It was used to illustrate that demographic characteristics between the groups were fairly similar, but it is now removed and the similarity is already evident from the descriptive data in the table.	**Methods**<br>Data analysis<br>~~Participant characteristics and demographics were compared between the ITT and PP groups using Pearson's chi-square test for categorical variables, with a p-value ≤ 0.05 used to indicate a statistically significant result.~~<br><br>**Results**<br>1st paragraph<br>~~No statistically significant differences in characteristics or demographics were found between the intervention group as a whole and those who followed the intervention protocol~~
and a number of the imputation decisions do not withstand careful scrutiny.	As a response to your comment we have revisited and clarified the description of data collection, data management and applied imputation strategies. Moreover we have applied a more conservative imputation strategy in the	**Methods**<br>Data collection<br>End of 2nd paragraph<br>Estimates of the time HCPs spent on checking the digital platform (e.g., logging in to the system three times daily and checking for messages) and writing messages during the study were based on interviews with the HCPs' team leader, who personally conducted most of these communications.<br><br>Data management

	scale up to 110 and 10 000 people, assuming that those never participating in the intervention have a time use equal of the mean value for actual time per patient. This was assumed to not underestimate time use. We have also extended the discussion on methodological limitations regarding imputation choices and rephrased a bullet point in the Strengths and limitations of this study section.<br><br>We have aimed to be as transparent as possible in reporting our imputation decisions. Our intention was to present the data in a way that allows readers to adjust or interpret the resource use estimates according to their own context and needs. To make this possible we have reported on both actual time per participant where data was available, and imputed time, in Table 4. We appreciate your feedback and would welcome specific suggestions.	Imputations were used to avoid underestimating the total time required for the intervention. The reasons for the missing data guided the imputation choices, which were agreed upon following discussions within the research team. The main reason for the missing data on the telephone support was a delay in implementing a more advanced data collection sheet. This sheet was designed to specify time use for various types of phone calls beyond health plan calls (e.g., introduction, technical support, and healthcare support calls). While the dates of these calls were documented from the outset of the study, their categorisation was conducted retrospectively for participants for whom the data sheet had not yet been developed, and time-use data had not been implemented. Details on the missing data, the applied imputation method, and the motives for the choices are provided in Appendix 3.<br><br>Calculations of the time use for HCPs were converted to units of patients per full-time employee and full-time employee per 10 000 patients, based on the assumption that 1880 hours constitute one full-time year.[15] We anticipated that approximately 10% of the work hours were spent on other tasks (e.g., education, meetings, and informal breaks). In calculations of the time use of HCPs we assumed that the time spent checking the digital platform was equal regardless of the number of participants (i.e., time used for taking action was measured separately).<br><br>**Results**<br>**See Table 4** (updated calculations, clarifications on imputed time)<br>**See Appendix 3**, added<br><br>HCPs spent most of their time on health plan calls with patients and documenting health plans, averaging 2.5 hours per patient, with the following breakdown: health plan calls (77 minutes), other phone calls (15 minutes), and documentation (50 minutes) (Table 4). The HCPs also spent approximately 78 hours per year checking the platform to assess whether further actions were needed (1.5 hours/week for all patients). Population size did not affect the time required to check the platform. Responding to messages from patients (n=110; 3 minutes/message) required an additional 19 hours. Translated into clinical practice, each HCP could provide the combined telephone and digital platform support to approximately 700 patients per year, equivalent to 15 full-time HCPs per 10 000 patients.

		**Discussion**<br>Strenghts and limitations<br><br>However, some methodological challenges were encountered in measuring time use. A major limitation was that the planned collection of login frequency and time required for activities from the digital platform could not be performed due to restrictions within in the portal. Instead, digital platform activities were manually collected through the platform, and time spent on the platform was self-reported, which may have introduced bias (e.g., memory or social desirability bias). Additionally, there were missing ratings of time spent on calls for technical and healthcare support for a small number of participants, primarily due to the initial communication lists not being fully developed at the trial's outset. To handle missing data, either expert ratings from HCPs conduction the trial were used, or imputation based on time reported for other patients. Mean imputation can be used when data are missing completely at random,[39] which was assumed here because the omissions were largely due to the delay in data collection at the start of the intervention. Despite the potential for bias resulting from this imputation method, can result in a bias[40], it was necessary in this instance to prevent an underestimation of total time. To avoid the narrowing effect on confidence intervals caused by mean imputation, the intervals were reported based on pre-imputation data, while the imputed data were only added in the final estimate of total time.<br><br>Strengths and limitations of this study<br>• Imputations were used ~~to correctly estimate~~ not to underestimate time use.
A final piece of advice to the authors is more clearly to distinguish their PROTECT trial from the very famous ProtecT three-arm RCT in prostate cancer.	Thank you for your comment. We appreciate the observation and understand the potential for confusion with the well-known ProtecT trial in prostate cancer. However, PROTECT is the established name of our study and has been used consistently in related publications and project	**Abstract**<br>Design<br>An exploratory, descriptive, observational study embedded in the “Person-centred care at a distance (PROTECT)” randomised controlled trial (ClinicalTrials.gov: NCT03183817) as part of a process evaluation.<br><br>**Introduction**<br>4th paragraph<br><br>This study is based on the randomised controlled trial (RCT) "Person-centred care at

	documentation. To avoid misunderstanding, we have added the full explanation of the acronym PROTECT in the abstract and clarified the context and scope of our PROTECT trial in the introduction to ensured that it is clearly distinguished from the ProtecT study.	a distance (PROTECT)", [24] which evaluates an intervention meeting the European standard for person-centred care[25].

VERSION 2 - REVIEW

**Reviewer**	**1**
**Name**	**Flink, Maria**
**Affiliation**	**Karolinska Institutet, Neurobiology, Care Sciences and Society**
**Date**	**03-Sep-2025**
**COI**	

Thank you for the thorough revision of the manuscript. My only remaining concern is the conclusion that 15 HCP could provide support to 10 000 patients. I lack some nuance regarding that patients participating in RCTs are not fully comparable to the normal population. In the Ali et al reference from 2021, the inclusion/exlusion criteria clearly points to that the intervention group is not the average population. I would suggest that you add a statement on this in your conclusion, and prefarably add the inclusion/exclusion critera also in this manuscript.

**Reviewer**	**2**
**Name**	**Hewison, Jenny**
**Affiliation**	**University of Leeds, Leeds Institute of Health Sciences**
**Date**	**05-Sep-2025**
**COI**	

The paper is substantially improved and I have only a few comments on the revised version.

Intro para 4: I still have residual concerns about the reporting of the composite primary outcome in the main trial. In my view it is misleading to say that 'the intervention had a

limited effect on the primary outcome in the PROTECT-trial', because by conventional criteria, there was no evidence of an effect, and that really does need to be made clear.

(From protocol paper: 'The primary end point will be the composite score of change in general self-efficacy and rehospitalisation or death 6 months after randomisation.')

A small drafting point: it would be better to say, 'Imputations were used in order not to underestimate time use.'

Results first line, and Tables 2 and 3: I still think it needs to be made clearer that one of these groups is in fact a subgroup of the other, so maybe the Results could say, '…comparable between the subgroup of participants who adhered to the intervention per protocol (n=76) and all individuals assigned to the intervention group according to the intention-to-treat principle (n=110), as detailed in Table 2'. The column titles in the tables could then use the same wording.

VERSION 2 - AUTHOR RESPONSE

**Reviewer: 1**<br>**Comments to the Author:**		
Thank you for the thorough revision of the manuscript. My only remaining concern is the conclusion that 15 HCP could provide support to 10 000 patients. I lack some nuance regarding that patients participating in RCTs are not fully comparable to the normal population. In the Ali et al reference from 2021, the inclusion/exlusion criteria clearly points to that the intervention group is not the average population. I would suggest that you add a statement on this in your conclusion,	Thank you for your thoughtful and thorough feedback. We appreciate your point regarding the generalizability of the conclusion that 15 healthcare professionals could support 10,000 patients. We agree that this estimate should be interpreted with caution, particularly given the applied inclusion and exclusion criteria in the randomized controlled trial. We have clarified this in the discussion and revised the conclusion at two places in the manuscript as recommended to clarify this.	**Discussion**<br>**Paragraph 4**<br>The ability to access health-supporting innovations when needed is essential in enabling preventative efforts. Both remote services and person-centred care are important support structures in working towards this vision.[2 3] In this study, the PROTECT intervention[24] served as a case for exploring the intervention costs of remote person-centred care to promote health and prevent health decline among individuals diagnosed with CHF or COPD. According to our findings, it appears that, based on the time distribution reported, it would be possible for 15 full-time HCPs to administer the intervention remotely to as many as 10 000 patients. The unique perspective gained from mapping time use and intervention engagement among both patients and HCPs can be used in strategic planning and decision-making processes, ultimately supporting the transition towards health-promoting and preventive services delivered through remote, person-centred care. When interpreting the results, it is essential to consider the characteristics of the study sample, which reflects the inclusion and exclusion criteria applied in the RCT …<br><br>**Conclusion**<br>This study provides insights into how usage patterns of a remote person-centred intervention differed between patients and HCPs. Although HCPs were accessible as needed, patients predominantly devoted their time to self-care activities. Given the reported time distribution in the PROTECT trial, a team of 15 full-time HCPs could co-create, follow up, and document health plans and be accessible for health

		support regarding these plans to 10 000 patients. This finding should be interpreted considering the characteristics of the study sample, which was determined by the inclusion and exclusion criteria applied in the RCT. While our observations indicate potential for scaling up remote person-centred care in preventive services, further investigation is needed to assess the effectiveness of these interventions in improving health outcomes. In addition, their costs should be evaluated within real-world settings and across more diverse patient populations.<br><br>**Abstract**<br>**Objectives:** To describe the usage patterns of patients and healthcare professionals (HCPs) using a person-centred telehealth and e-health intervention.<br>**Design:** An exploratory, descriptive, observational study embedded in the “Person-centred care at a distance (PROTECT)” randomised controlled trial (ClinicalTrials.gov: NCT03183817) as part of a process evaluation. Data on intervention use and time spent on the intervention were collected. Descriptive statistics were calculated.<br>**Setting:** Participants were recruited from nine public primary healthcare facilities located in various areas of Gothenburg, Sweden. **Participants:** 110 patients participating in the intervention group in the PROTECT trial were included. Participants were diagnosed with chronic heart failure (CHF, n=42), chronic obstructive pulmonary disease (COPD, n=56), or both (n=12). They were 33–93 years old (mean 71 years).<br>**Primary and Secondary Outcome Measures:** ~~This is~~ A secondary outcome report on resource use.<br>**Intervention:** The 6-month-long intervention was performed as an add-on to standard care and comprised person-centred telephone support and access to a digital platform. Per-protocol use included co-creation of a health plan via the telephone and use of the digital platform at least once. Forms of use were tailored to the preferences and needs of the patients.<br>**Results:** Most intervention activities took place in the first 3 months of the intervention. Most patients used a combination of phone and digital support, spending most of their time using the digital platform. Overall, patients and HCPs spent 6 and 2.5 hours/patient using the intervention, respectively. Of this time, 1.5 hours involved synchronous communication through phone calls, with health-plan calls averaging 77 minutes.<br>**Conclusions:** The intervention usage patterns of patients and HCPs differed. Despite HCPs being accessible when required, patients dedicated most of their time to self-care practices. Based on time

		distribution data, 15 full-time HCPs could potentially co-create, document and follow up on health plans for 10 000 patients under study conditions.
and prefarably add the inclusion/exclusion critera also in this manuscript.	We have added the inclusion and exclusion criteria in the RCT in the methods section.	**Methods**<br>**Participants**<br>The PROTECT study included 222 patients diagnosed with CHF and/or COPD (112 in the control group and 110 in the intervention group). The present study comprises all patients (n=110) allocated to the intervention group. Additional inclusion criteria in the main trial were being listed at one of the participating primary care centres, understanding written and spoken Swedish, and having access to a device with an internet connection. Applied exclusion criteria were: severe impairment (e.g., cognitive) preventing the use of the e-health support, lack of a registered address, expected survival of less than 12 months, an ongoing documented diagnosis of alcohol or drug abuse, other conditions that could interfere with follow-up, and participation in another conflicting study[24].
**Reviewer: 2**<br>**Comments to the Author:**		
The paper is substantially improved and I have only a few comments on the revised version.	Thank you for acknowledging the improvements in the revised manuscript. We greatly appreciate the constructive feedback and specific suggestions provided during the review process, which have significantly contributed to strengthening the clarity and quality of the paper. We have addressed the remaining comments as per your suggestions.	
Intro para 4: I still have residual concerns about the reporting of the composite primary outcome in the main trial. In my view it is misleading to say that 'the intervention had a limited effect on the primary outcome in the PROTECT-trial', because by conventional criteria, there was no evidence of an effect, and that really does need to be made clear.<br>(From protocol paper: 'The primary end point will be the composite score of change in	We have rephrased paragraph 4 in the introduction to clarify that there was no intervention effect on the primary outcome.	While there was no effect on the primary outcome (a composite score of change in general self-efficacy and re-hospitalisation or death 6 months after randomisation) in the PROTECT-trial[26] patients perceived the intervention as a meaningful, accessible, and appreciated support for initiating or maintaining self-management activities.[9 27]

general self-efficacy and rehospitalisation or death 6 months after randomisation.')		
A small drafting point: it would be better to say, 'Imputations were used in order not to underestimate time use.'	We have rephrased the sentence as recommended in two places in the manuscript.	**Strengths and limitations of this study**<br>• Collected intervention use data from both patients and health care professionals.<br>• Detailed communication lists were regularly checked for accuracy.<br>• ~~Imputations were used not to underestimate time use.~~ Imputations were used in order not to underestimate time use.<br>• Self-reported time on digital platform activities due to limited automatic registration.<br>• No assessment of impact on time use for self-management except on the digital platform.<br><br>**Data management**<br>~~Imputations were used to avoid underestimating the total time required for the intervention.~~ Imputations were used in order not to underestimate time use.
Results first line, and Tables 2 and 3: I still think it needs to be made clearer that one of these groups is in fact a subgroup of the other, so maybe the Results could say, '…comparable between the subgroup of participants who adhered to the intervention per protocol (n=76) and all individuals assigned to the intervention group according to the intention-to-treat principle (n=110), as detailed in Table 2'. The column titles in the tables could then use the same wording.	We have clarified that the per-protocol group is a subgroup of the ITT group by applying the suggested rephrasing.	Demographic characteristics were comparable between the subgroup of participants who adhered to the intervention per protocol (n=76) and all individuals assigned to the intervention group according to the intention-to-treat principle (n=110), as detailed in Table 2.
